# Microneedle Mediated Iontophoretic Delivery of Tofacitinib Citrate

**DOI:** 10.1007/s11095-022-03190-5

**Published:** 2022-02-16

**Authors:** Amruta A. Dandekar, Harsha T. Garimella, Carrie L. German, Ajay K. Banga

**Affiliations:** 1 Center for Drug Delivery Research, Department of Pharmaceutical Sciences, College of Pharmacy, Mercer University, 3001 Mercer University Drive, Atlanta, GA 30341, USA; 2 CFD Research Corporation, 701 McMillian Way NW, Huntsville, AL 35806, USA

**Keywords:** Iontophoresis, Microneedles, Psoriasis, Tofacitinib citrate, Transdermal delivery

## Abstract

**Purpose:**

To investigate *in vitro* transdermal delivery of tofacitinib citrate across human skin using microporation by microneedles and iontophoresis alone and in combination.

**Methods:**

*In vitro* permeation studies were conducted using vertical Franz diffusion cells. Microneedles composed of polyvinyl alcohol and carboxymethyl cellulose were fabricated and successfully characterized using scanning electron microscopy. The microchannels created were further characterized using histology, dye binding study, scanning electron microscopy, and confocal microscopy studies. The effect of microporation on delivery of tofacitinib citrate was evaluated alone and in combination with iontophoresis. In addition, the effect of current density on iontophoretic delivery was also investigated.

**Results:**

Total delivery of tofacitinib citrate via passive permeation was found out to be 11.04 ± 1 μg/sq.cm. Microporation with microneedles resulted in significant enhancement where a 28-fold increase in delivery of tofacitinib citrate was observed with a total delivery of 314.7±33.32 μg/sq.cm. The characterization studies confirmed the formation of microchannels in the skin where successful disruption of stratum corneum was observed after applying microneedles. Anodal iontophoresis at 0.1 and 0.5 mA/sq.cm showed a total delivery of 18.56 μg/sq.cm and 62.07 μg/sq.cm, respectively. A combination of microneedle and iontophoresis at 0.5 mA/sq.cm showed the highest total delivery of 566.59 μg/sq.cm demonstrating a synergistic effect. A sharp increase in transdermal flux was observed for a combination of microneedles and iontophoresis.

**Conclusion:**

This study demonstrates the use of microneedles and iontophoresis to deliver a therapeutic dose of tofacitinib citrate via transdermal route.

## Introduction

Psoriasis is a chronic immune mediated inflammatory skin disease affecting a large population with an estimated 60 million people in 2021 [1]. It is often associated with several comorbidities, including psoriatic arthritis, psychological, cardiovascular, and hepatic diseases. Mild psoriasis is usually treated by topical treatment, but moderate to severe psoriasis needs phototherapy, conventional systemic therapies, or the use of biologics [2]. Although several drugs are approved to treat psoriasis, toxicity and failure rates associated with conventional systemic therapies and decreased efficacy over time in biologics underline the importance of alternative treatment options for psoriasis [3, 4]. An alternative approach involves developing inexpensive, easy to administer small-molecule agents that inhibit intracellular proteins or enzymes in the immune response. Janus kinase (JAK) inhibitors are one such class of drugs currently under investigation for the treatment of psoriasis [2]. Tofacitinib is a widely studied JAK inhibitor for treating psoriasis and is currently approved for rheumatoid arthritis, psoriatic arthritis, and ulcerative colitis [5]. It is proven effective against chronic plaque psoriasis in phase III clinical trials with an oral dose of 10 mg twice daily [6], making it a promising candidate for the treatment of psoriasis.

Existing marketed formulations of tofacitinib are oral tablets of its salt form- tofacitinib citrate. However, oral administration of tofacitinib citrate is associated with several side effects, including gastrointestinal disorders, upper respiratory tract infections, headache, and cold-like symptoms [7]. In addition, in 2019, a study indicated a higher risk of blood clots with oral administration of tofacitinib citrate, raising safety concerns [8]. Thus, alternative routes, more specifically localized routes, are being explored for delivery of tofacitinib citrate. A transdermal system that can bypass hepatic metabolism and provide local delivery with decreased side effects arising from systemic exposure can serve as a better delivery route for tofacitinib citrate.

Skin, with a large surface area of 1-2 m^2^ and ease of accessibility, is a possible route with high potential for administration of drugs [9]. However, stratum corneum, the outermost layer of skin, is a major barrier for penetration of drugs into and across skin. An ideal drug for passive diffusion is an unionized, potent, low molecular weight (less than 500 Da), moderately lipophilic (log P ~1-3) molecule with a melting point of < 250 °C [10]. Thus, passive delivery of hydrophilic or large molecules is difficult. Various physical enhancement technologies such as microporation by microneedles, iontophoresis, electroporation, and sonophoresis have been investigated to deliver such molecules [11, 12]. The hydrophilic nature and positive charge at a physiological pH of 7.4 make passive delivery of tofacitinib citrate a challenge [13]. Literature also reports slower penetration and low permeability of tofacitinib citrate across the skin [7]. Therefore, in this study, we investigated the effect of physical enhancement techniques, namely microneedles and iontophoresis alone and in combination for transdermal delivery of tofacitinib citrate.

Microneedles are micron-sized needles that create hydrophilic microchannels in the skin bypassing the stratum corneum layer enabling delivery of hydrophilic drugs [14]. These microneedles offer a pain-free, needle-free mode of drug delivery. Our lab has successfully delivered glycopyrrolate, cimetidine, vismodegib, donepezil using microneedles [15-18]. Polyvinyl alcohol (PVA) and carboxymethyl cellulose (CMC) are hydrophilic biocompatible polymers reported to be used for microneedle fabrication [19, 20]. We fabricated PVA-CMC microneedles to microporate skin, which was investigated for microneedle-mediated transdermal delivery of tofacitinib citrate.

Iontophoresis is another technique that helps in delivering neutral and charged drug molecules using a small, physiologically acceptable amount of electric current (0.5 mA/cm^2^ or less) [21]. Iontophoretic delivery of several drugs has been reported [10]. Electro-osmosis and electrorepulsion are the two driving forces enabling iontophoretic delivery of molecules where current densities and duration of application can be altered for modulated delivery [21]. Our study investigates iontophoretic delivery of tofacitinib citrate at two current densities.

Further, the combination of microneedles and iontophoresis has been explored previously for a wide range of molecules, including small molecules [22, 23], peptides [24], and proteins [25], to investigate any synergistic effect observed as both techniques work by different mechanisms and the effect is dependent on various factors. Therefore, we investigated the combination of microneedle and iontophoresis to assess the effect of combination on delivery of tofacitinib citrate. Transdermal delivery of tofacitinib citrate using hollow and dissolving microneedles was investigated recently [26]. However, iontophoretic delivery of tofacitinib citrate and its effect when combined with microneedles has not been investigated previously. Thus, this study focuses on transdermal delivery of tofacitinib citrate into and across full-thickness human skin using microneedles and iontophoresis alone and in combination. The effect of microporation was further characterized using electrical resistance measurement, dye binding studies, scanning electron microscopy (SEM), confocal microscopy, and histological evaluation.

## Materials and methods

### Materials

Tofacitinib citrate was purchased from CSN Pharm (Arlington Heights, IL, U.S.A). Phosphate buffered saline, pH 7.4 (PBS, 10X), sodium phosphate dibasic, orthophosphoric acid, and silver wire (0.5 mm diameter, 99.99%) were obtained from Fisher Scientific (Fair Lawn, NJ, U.S.A). HPLC grade acetonitrile and methanol were procured from Pharmco-Aaper (Brookfield, CT, U.S.A). Polyvinyl alcohol (PVA 26-88 EMPROVE® exp Ph Eur, USP, JPE) and carboxymethyl cellulose sodium salt (CMC low viscosity CAS 9004-32-4) were obtained from Sigma Aldrich (St. Louis, MO, U.S.A). Silver chloride electrodes (2 mm x 4mm) were purchased from A-M systems (Sequim, WA, U.S.A.). Staining solutions, namely methylene blue and Fluoresoft (0.35%), were procured from Eastman Kodak Co. (Rochester, NY, U.S.A) and Holles Laboratories Inc. (Cohasset, MA, U.S.A), respectively. Full-thickness human skin tissue was procured from the National Disease Research Interchange (NDRI) with support from NIH grant U42OD11158.

### Methods

#### Solubility studies

Saturation solubility of tofacitinib citrate was determined in 1X PBS solution. To test saturation solubility, excess amount of drug was added to 1mL of solvent followed by overnight shaking at room temperature. After that, the solution was centrifuged at 13,400 rpm for 15 min at 25 °C, filtered through 0.22 μm syringe filters (Cell treat Scientific Products, Shirley, MA, USA), diluted appropriately, and analyzed using HPLC.

#### Fabrication of PVA-CMC microneedles

Microneedles were fabricated by micromolding technique. First, the polydimethylsiloxane molds were prepared by the previously optimized method [27]. Then, to fabricate microneedles, 3 g of PVA and 1 g of CMC sodium salt were dissolved in 10 mL of DI water heated to 90 °C, and the mixture was kept in the oven at 90 °C until the polymers dissolved. The dissolved polymeric solution (300 μL) was then placed into the PDMS mold, centrifuged (Eppendorf Centrifuge 5810 R, Eppendorf AG, Hamburg, Germany) at 4000 rpm for 15 mins at 40 °C to pull the solution into cavities without formation of air bubble. The microneedles were then dried in a convection oven at 60 °C for three days, separated from the mold, and stored in a desiccator until use.

#### Skin preparation

Fresh frozen full-thickness human cadaver skin tissue from multiple sites (abdomen, calf and back) and donors was obtained from National Disease Research Interchange (NDRI). The subdermal fat tissue was removed using forceps and scissors, and the skin tissue was stored at −80 °C until further use. At the time of permeation testing, the frozen skin tissue was thawed in 1X PBS at 37°C. Then, using die punches (diameter = 22 mm, area = 3.8 cm^2^), the skin tissue was cut into circular pieces and mounted on the Franz diffusion cell for permeation studies [28]. The thickness of each piece was recorded using a thickness gauge (MTG-DX2 by Checkline®, Cedarhurst, NY, USA). The average thickness of skin used for permeation studies was 1450 ± 186 μm.

#### Skin integrity measurement

The barrier integrity of skin was assessed by measuring skin’s electrical resistance. A digital multimeter (Agilent Technologies, Santa Clara, CA, U.S.A), a waveform generator connected to a silver/silver chloride electrode was used for this purpose. First, the skin piece was mounted on a Franz cell containing 5 mL of 1X PBS in the receptor compartment. Then, 300 μL of 1X PBS was added to the donor chamber, and the skin tissue was kept for equilibration for 15 mins. After that, the silver wire was placed in the receptor compartment, and the silver chloride electrode was placed in the donor compartment (without touching the skin). Next, the voltage drop across the circuit (Vo) and skin (Vs) was recorded using the multimeter. The resistance was calculated according to the following equation [18]:

$$R_{S}=V_{S}R_{L}∕\left(V_{O}-V_{S}\right)$$

Where the load resistor (R_L_) was 100 KΩ and *V_O_* was set to 100 mV. Resistance values were calculated in KΩ. Skin samples having a resistance greater than 10 KΩ were used for permeation study [29].

#### In vitro permeation testing (IVPT)

IVPT was performed using vertical static Franz diffusion cells (PermeGear, Hellertown, PA, U.S.A) having a diffusion area of 0.64 cm^2^. The selected (treated/untreated with microneedles) skin pieces were clamped between the donor and receptor compartment, ensuring no air bubble formation. The receptor compartment was filled with 5 mL of 1X PBS, selected as a receptor solution based on solubility studies to maintain the sink condition. The receptor compartment was maintained at 37 °C using a recirculatory water bath to keep the skin temperature at 32 °C. After putting the drug solution in the donor chamber, receptor samples (300 μL) were taken at 0,1,2,4,8, 22, and 24 h from the sampling arm, followed by replenishing the receptor solution with an equal volume of 1X PBS at each time point. At the end of the study, two dry cotton swabs were used to remove excess donor solution. After that, the donor chamber was removed, and the permeation area was gently wiped with a cotton swab dipped in 5% lauryl ether sulfate for 30s in a circular motion to remove the unabsorbed drug from skin surface. This process was repeated once again. Finally, a new donor chamber was placed, and the permeation area was washed with 1 mL of 1X PBS thrice. The skin piece was then dried using two dry cotton swabs and removed from the Franz diffusion cell for skin extraction study. All samples were analyzed using HPLC, and results were reported as mean ± SE (n=4). The specific protocol followed for each group is as follows:

#### Passive permeation

For testing passive diffusion of tofacitinib citrate across skin, 100 μL of 5.9 mg/mL (90% saturation solubility in 1X PBS) tofacitinib citrate solution in 1X PBS was added in the donor compartment, and IVPT study was conducted as mentioned earlier.

#### Microneedle mediated delivery

To assess microneedle-mediated delivery of tofacitinib citrate, the skin piece was microporated using the fabricated PVA-CMC microneedles. First, the skin piece was placed on parafilm (Parafilm “M” Laboratory film, Neenah, WI, USA) to mimic the soft tissue beneath the skin, thereby avoiding excess pressure on the skin. Next, the PVA-CMC microneedles (10 x 10 array of 100 microneedles; 395 μm needle length) were applied perpendicularly with equal force to the skin using fingers for 2 min to ensure uniform microporation. After application, the microneedle array was removed, and the skin piece was mounted back on the Franz diffusion cell, ensuring that the diffusion area matched the microporated area. Finally, the donor compartment was filled with 500 μL of 5.9 mg/mL tofacitinib citrate solution in 1X PBS followed by the same protocol mentioned in IVPT testing. Skin microporation was further characterized using dye binding, histology, SEM, and confocal microscopy.

#### Iontophoretic delivery

As tofacitinib citrate is positively charged at pH 7.4, anodal iontophoresis was performed. The iontophoretic setup consisted of a silver wire (anode) placed in the donor chamber and silver chloride (cathode) in the receptor chamber. These electrodes were connected in series to a source of constant current supply device (Keithley 2400 Source Meter R, Keithley Instruments Inc., Cleveland, OH, USA). The required current density was applied for 4 h, followed by passive delivery to 24 h. The donor chamber contained 500 μL of 5.9 mg/mL tofacitinib citrate solution in 1X PBS. Two different current densities (0.1 and 0.5 mA/cm^2^) were used to study the effect of current density on delivery of tofacitinib citrate, with the rest of the protocol being same as explained previously.

#### Delivery using combination of microneedle and iontophoresis

To test delivery of tofacitinib citrate using a combination of microneedle and iontophoresis, the skin piece was microporated using fabricated microneedles, followed by iontophoretic delivery using the setup mentioned in iontophoretic delivery section. The combination was tested at a current density of 0.5 mA/cm^2^ using the same IVPT protocol.

#### Skin extraction / distribution study

To determine the amount of tofacitinib citrate delivered into skin, an extraction study was performed. Following IVPT, skin permeation area was cut out and added to a tube along with 3 mL of methanol. The skin tissue was then homogenized using a bead mill homogenizer (Bead Ruptor 24 Elite by Omni International, Kennesaw, GA, U.S.A) at set parameters (number of cycles: 2, speed: 6.00 m/s, time: 30 s, dwell time: 60 s) followed by 4 h of shaking and then filtered through 0.22 μm syringe filters (Cell treat Scientific Products, Shirley, MA, USA) and analyzed using the HPLC method. Results were reported as mean ± SE (n=4) for each group.

#### Lag time and flux calculation

A graph of the cumulative amount of tofacitinib citrate delivered per unit area was plotted against time to determine the lag time required for permeation of tofacitinib citrate into the receptor. The X-intercept of the linear portion was used to determine the lag time. The flux profile was plotted with amount of tofacitinib citrate delivered into the receptor per unit area per unit time.

#### Quantitative analysis

Receptor and skin extraction samples were analyzed using a validated UV-HPLC method. Isocratic reverse phase chromatographic analysis was performed using Waters Alliance 2695 separation module (Milford, MA, USA) coupled with 2996 photodiode array detector. The separation was carried out on Phenomenex C18 (250 x 4.6 mm, 5μm) maintained at 25 °C. The mobile phase consisted of acetonitrile and pH 7 buffer (10 mM Na_2_HPO_4_) in the ratio of 25:75 with a 1 mL/min flow rate and an injection volume of 10 μL. The run time was 15 minutes, the retention time was 7 minutes, and the detection was carried out at 286 nm. The limit of detection and quantification were 0.03 μg/mL and 0.08 μg/mL respectively, whereas the linearity range was 0.1-50 μg/mL (R^2^= 1). No interference due to components leaching out from skin was observed for quantification of tofacitinib citrate.

#### Data Analysis

All results were reported as mean (n=4) with standard error (SE) or standard deviation (SD). GraphPad Prism (GraphPad Software, San Diego, CA; version 8.4.3) was used for statistical analysis. One-way ANOVA with Tukey’s post-hoc test and Student’s *t*-test were used for comparison, and a *p*-value of less than 0.05 was considered to conclude a significant difference between the test groups.

#### SEM study

The structure and morphology of microneedles and microchannels created by microneedles were investigated using the Phenom™ field emission SEM system (Nanoscience Instruments, Inc., Phoenix, AZ, USA). Microneedles were mounted directly on metal stub whereas, for microchannels, full-thickness human skin was treated with microneedles for 2 mins, dried, and then mounted on metal stub using a double-sided tape (Ted Pella, Inc. Redding, CA, U.S.A.). All samples were examined using field emission S.E.M. (Hitachi, S4100).

#### Dye binding study

The microchannels created by microneedles were confirmed by a dye binding study using a 1% methylene blue solution. First, full-thickness human skin was placed on parafilm and treated with microneedles. After removal of microneedle array, 1% methylene blue solution was applied to the microneedle treated area for 2 min. Then, the excess dye was removed using Kimwipes and alcohol swabs, and the stained microchannels were visualized.

#### Confocal laser scanning microscopy (CLSM) study

To determine the depth of microchannels created by microneedles, CLSM studies were carried out (n=3). Fluoresoft® (0.35%, 200 μl) was applied to full-thickness human skin treated with the microneedles for 1 min. Excess calcein was removed, and the skin sample was placed on the glass slide and visualized using a computerized Leica SP8 confocal laser microscope. Samples were viewed with a 10X objective at an excitation wavelength of 496 nm. X-Z sectioning was employed using Application Suite-Advanced Fluorescent software (step size = 15 μm) by Leica to determine the depth of microchannels.

#### Histology studies

Histological studies were used to characterize further the microchannels created by microneedles using a similar procedure as mentioned in previous studies [30]. Full-thickness human skin treated with microneedles was fixed flat in Tissue-Tek using an optical coherence tomography compound (OCT) medium and stored at −80 °C to solidify the block. Microm HM 505 E (Southeast Scientific, Inc., GA, USA) was used to section the block where the section thickness was 10 μm. The sectioning was performed at −20 °C with the block glued to the object holder in the cryotome chamber. The sections were fixed on a glass slide (Globe Scientific, Inc., NJ, USA), stained with hematoxylin and eosin, and viewed under Leica DM 750 microscope (Leica microsystems, Buffalo Grove, IL, USA).

## Results

### Solubility studies

The solubility of tofacitinib citrate in 1X PBS was found to be 5.9 mg/mL. The solubility was enough to maintain sink conditions, and hence 1X PBS was selected as receptor solution.

### Characterization of microneedles and microchannels

#### SEM of fabricated microneedles and microchannels

**Fig. 1:**
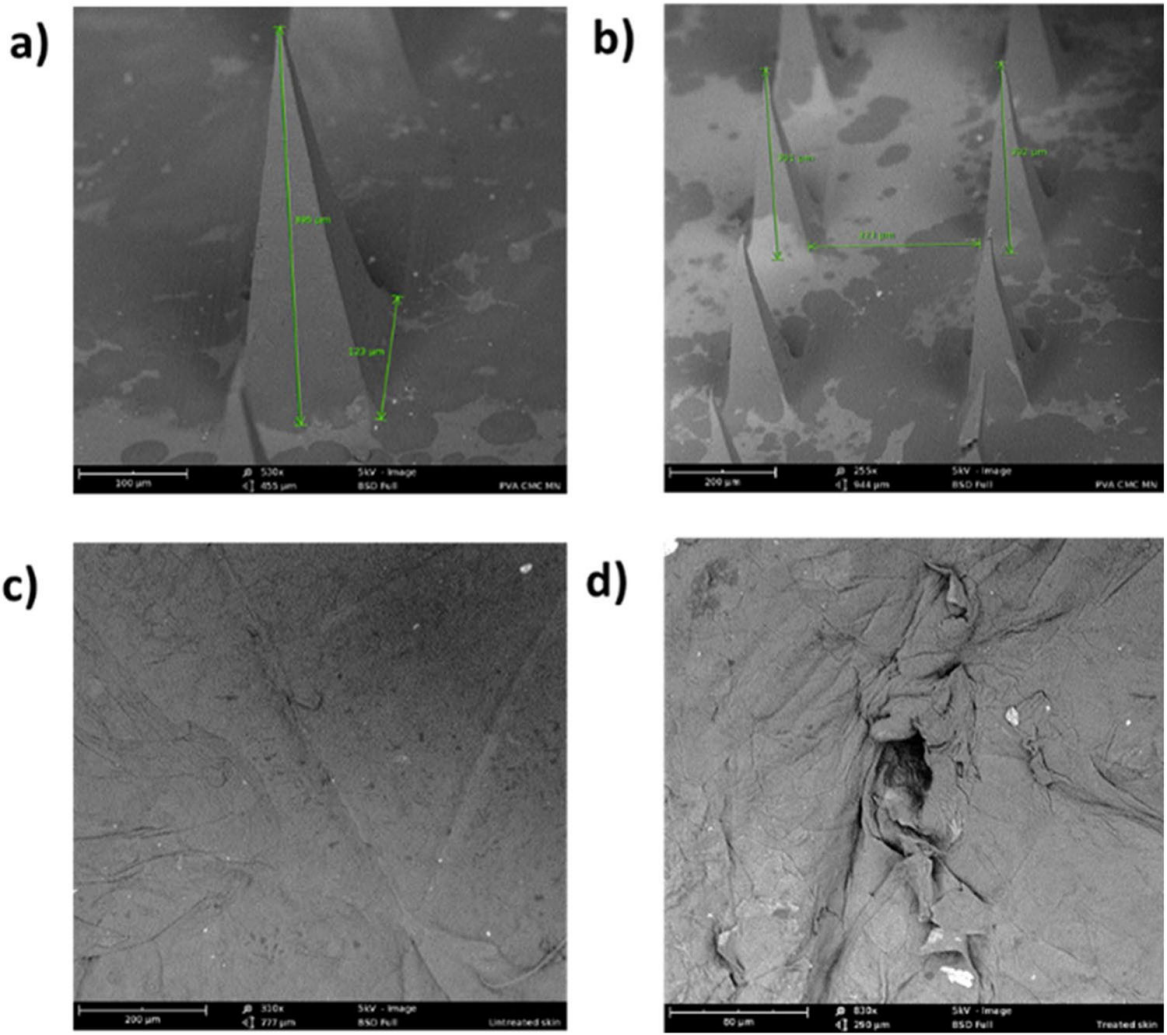
(a and b) SEM image of fabricated PVA-CMC microneedles (c) SEM image of untreated skin (d) SEM image of microneedle treated skin

SEM image of fabricated PVA-CMC microneedles showed pyramidal-shaped microneedles having tip to base length of 395 ± 2.6 μm, base of approximately 123 μm, and needle-to-needle distance of 323 μm (Fig. 1a and b). The fabricated microneedles had sufficient strength to create micropores without breaking. Figure 1c shows an SEM image of untreated skin with intact stratum corneum. Fabricated microneedles successfully microporated skin, creating microchannels that are visible as micropores on the skin surface. Figure 1d shows a magnified image of a single microchannel.

#### Dye binding and histology studies

**Fig. 2:**
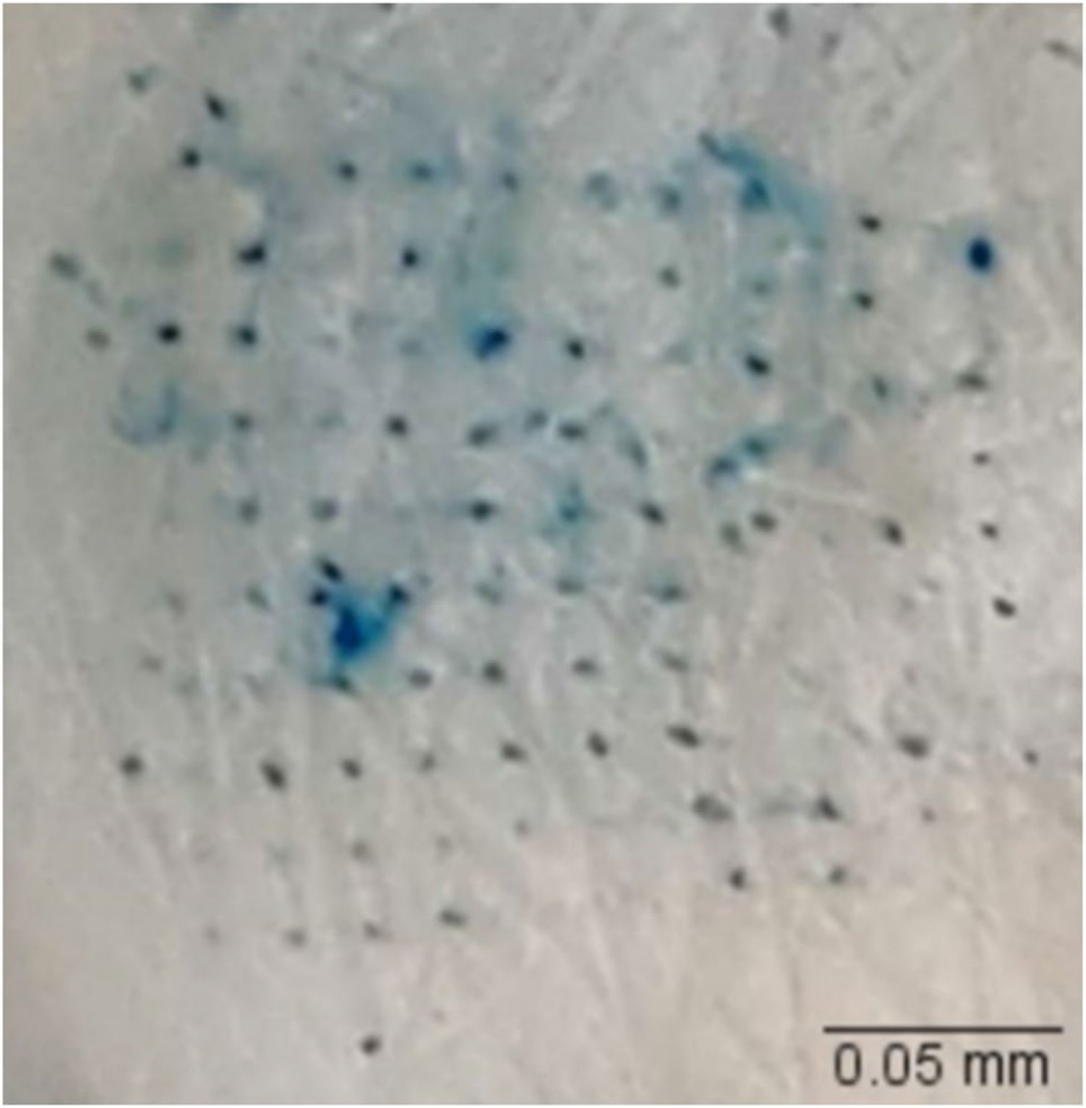
Methylene blue-stained microchannels in skin

The sharpness, density, and penetration ability of fabricated microneedles were tested using a dye binding study. Skin treated with microneedles (Fig. 2) showed microchannels stained with hydrophilic methylene blue dye, confirming skin microporation.

**Fig 3:**
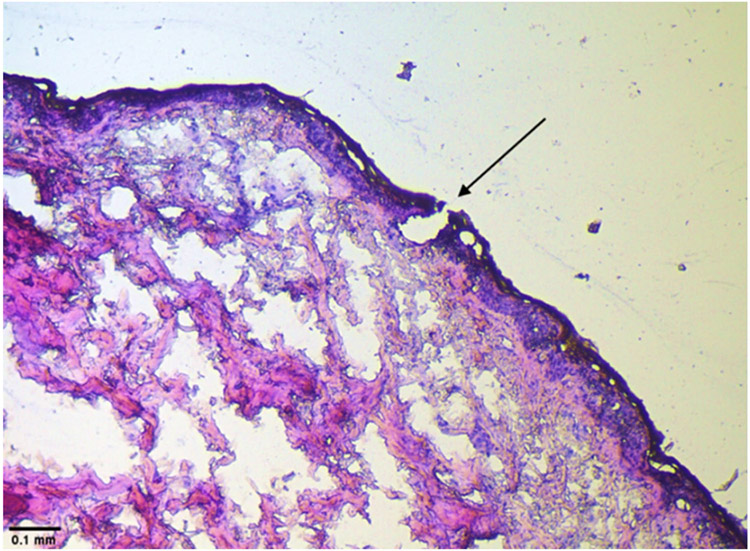
Histological evaluation of skin treated with microneedles showing formation of microchannel

The morphology of microchannels created by microneedles was visualized by performing histological characterization of skin. The vertical section of skin treated with microneedles showed disruption of stratum corneum, as seen in Fig. 3, indicating microchannel formation.

#### CLSM studies

**Fig 4:**
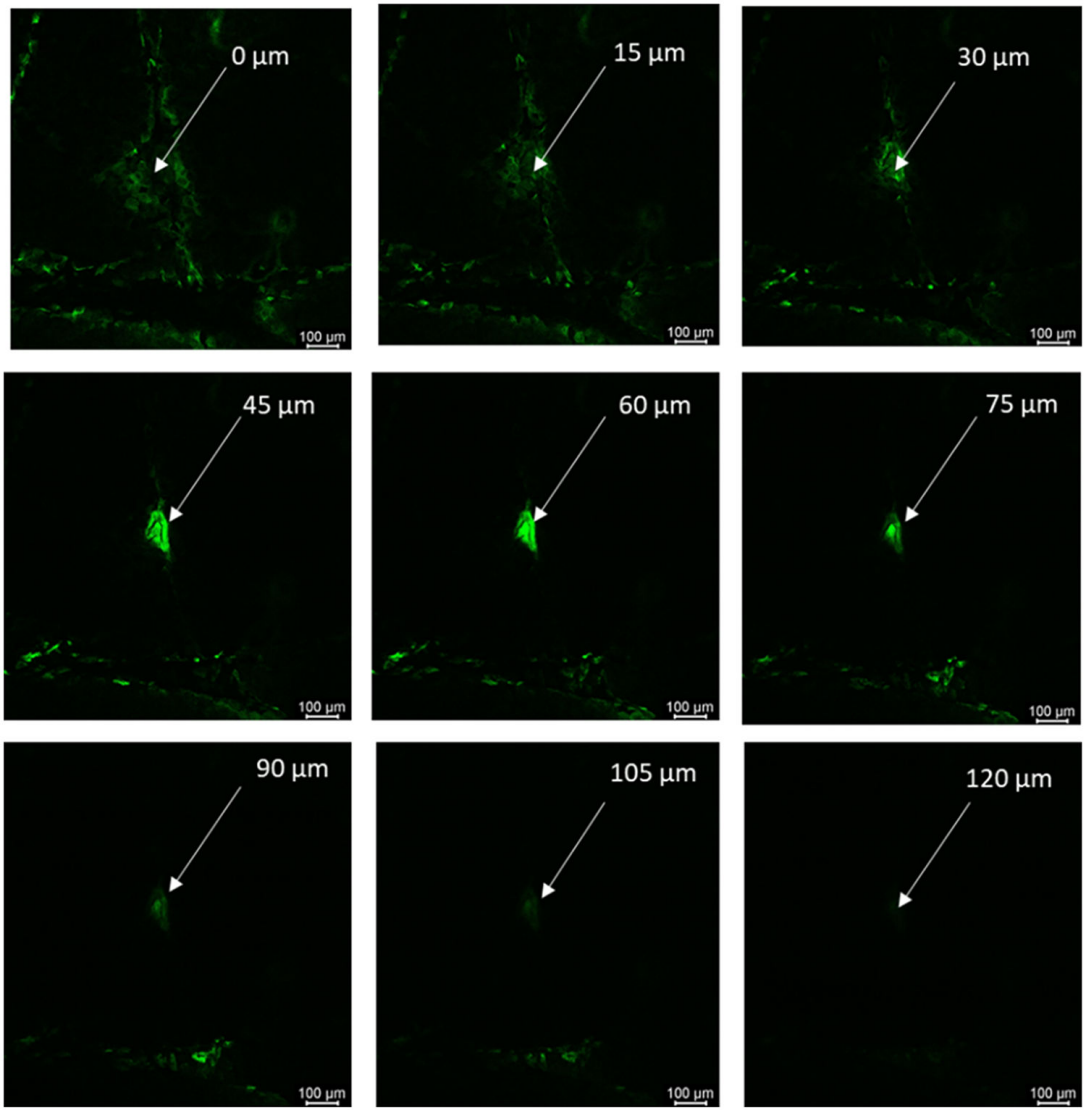
Confocal microscopy image showing z-stack of microchannel created by microneedles in full-thickness human skin

The depth of micropores created by microneedles was determined using CLSM studies. Micropores stained with fluorescent dye confirmed the formation of microchannels in skin. Next, the depth of micropores was calculated by performing a z-stack where the stained microchannel was imaged at different depths (z) until the fluorescent dye was no longer visible. Figure 4 shows the image of the z-stack. The depth of pore created by microneedles was estimated to be 120 ± 15 μm.

### *In vitro* permeation testing

#### Passive permeation

**Fig. 5:**
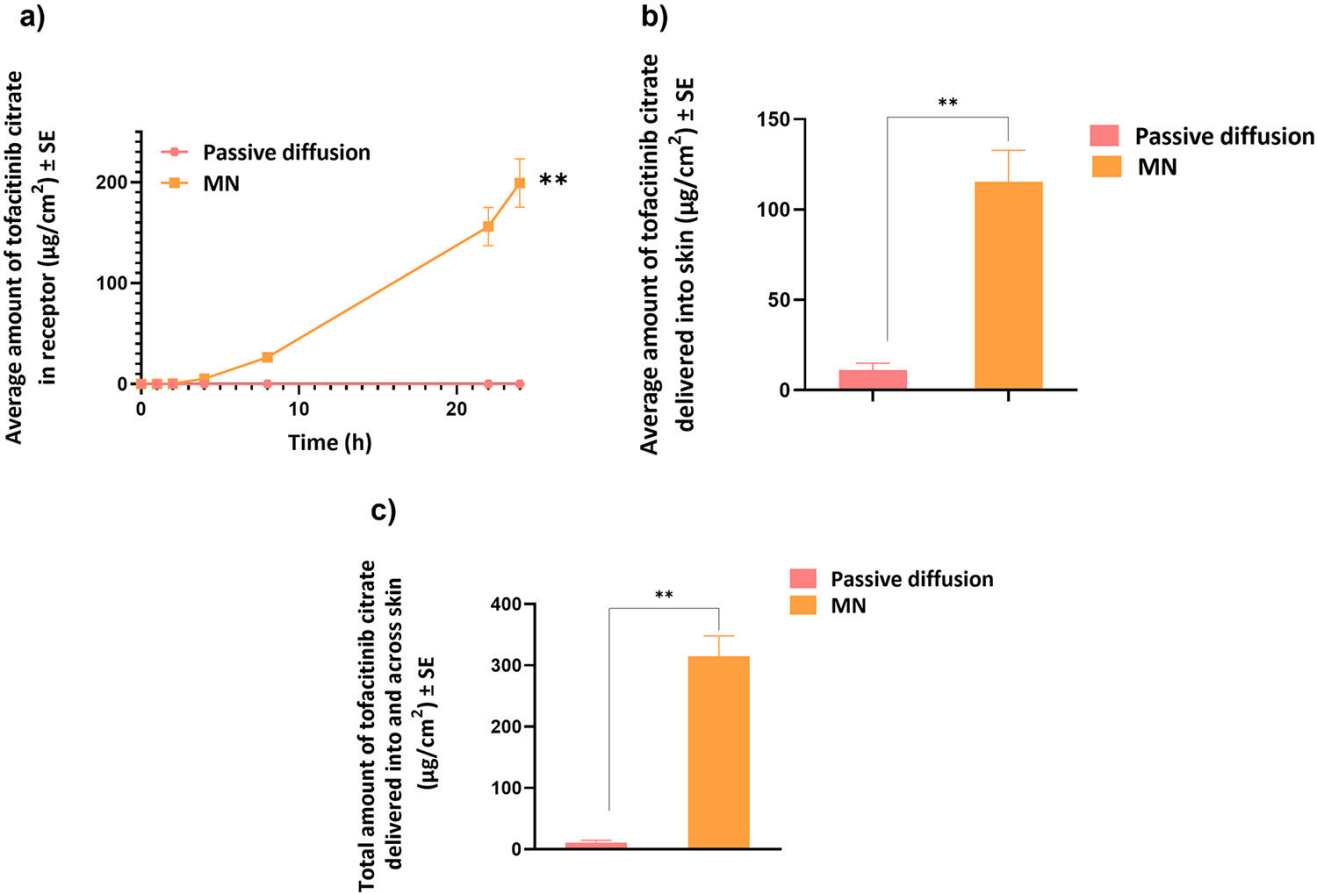
Microneedle mediated delivery: (a) Permeation profile of tofacitinib citrate across full-thickness human skin into receptor over 24 h (b) Amount of tofacitinib citrate delivered into skin at the end of 24 h (c) Total amount of tofacitinib citrate delivered into and across full-thickness human skin. Data presented as average values ± SE (n=4); **: p < 0.01; MN: Microneedles

There was no passive diffusion of tofacitinib citrate across full-thickness human skin into the receptor chamber (Fig. 5a). The amount delivered into skin at the end of 24 h was 11.04 ± 1 μg/cm^2^. This indicated low passive permeability of tofacitinib citrate.

#### Microneedle mediated delivery

The effect of microporation on transdermal delivery was investigated by testing delivery of tofacitinib citrate across skin treated with PVA-CMC microneedles. Creation of microchannels was supported by the results of several characterization studies, including dye binding, SEM, CLSM, and histology, as described earlier. Microporation resulted in a significantly higher delivery of tofacitinib citrate both across (199.32 ± 24 μg/cm^2^) and into (115.38 ± 17.5 μg/cm^2^) skin with a total delivery of 314.7 ± 33.3 μg/cm^2^ (p<0.01) as seen in Fig. 5. The lag time was found to be 3.7 h.

#### Iontophoretic delivery

**Fig. 6:**
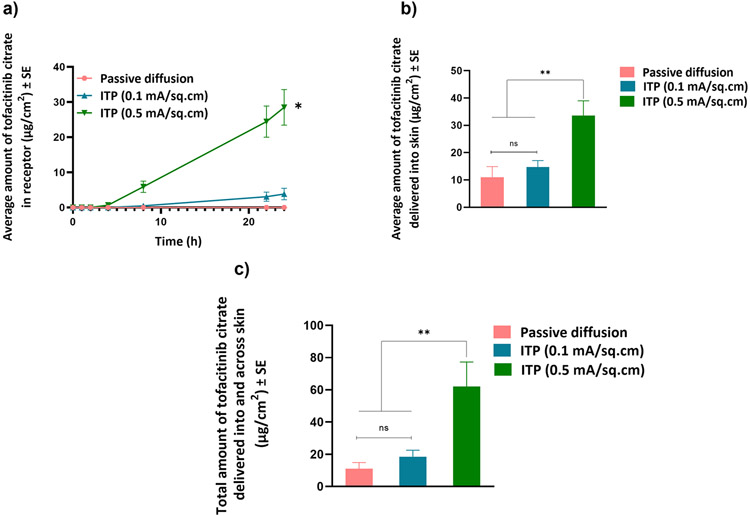
Iontophoretic delivery: (a) Permeation profile of tofacitinib citrate across full-thickness human skin into receptor over 24 h (b) Amount of tofacitinib citrate delivered into skin at the end of 24 h (c) Total amount of tofacitinib citrate delivered into and across full-thickness human skin. Data presented as average values ± SE (n=4); *: p < 0.05, **: p < 0.01; ITP: Iontophoresis

As tofacitinib citrate is positively charged at pH 7.4, anodal iontophoresis was performed and the delivery was tested at two different current densities, 0.1 and 0.5 mA/cm^2^. Iontophoresis at 0.5 mA/cm^2^ delivered significantly higher total amount of tofacitinib citrate (62.07±7.6 μg/cm^2^) as compared to both iontophoresis at 0.1 mA/cm^2^ (18.56±4 μg/cm^2^) and passive diffusion. There was no significant difference in total delivery of tofacitinib citrate between passive diffusion and iontophoresis at a current density of 0.1 mA/cm^2^. Figure 6 shows delivery of tofacitinib citrate into and across skin for all three groups. Based on the highest delivery, 0.5 mA/cm^2^ was selected as current density for testing delivery of tofacitinib citrate using a combination of microneedles and iontophoresis. The observed lag time for permeation of tofacitinib citrate into receptor at the current density of 0.1 and 0.5 mA/cm^2^ was 6.1 h and 3.7 h, respectively.

#### Delivery using a combination of microneedles and iontophoresis

**Fig. 7:**
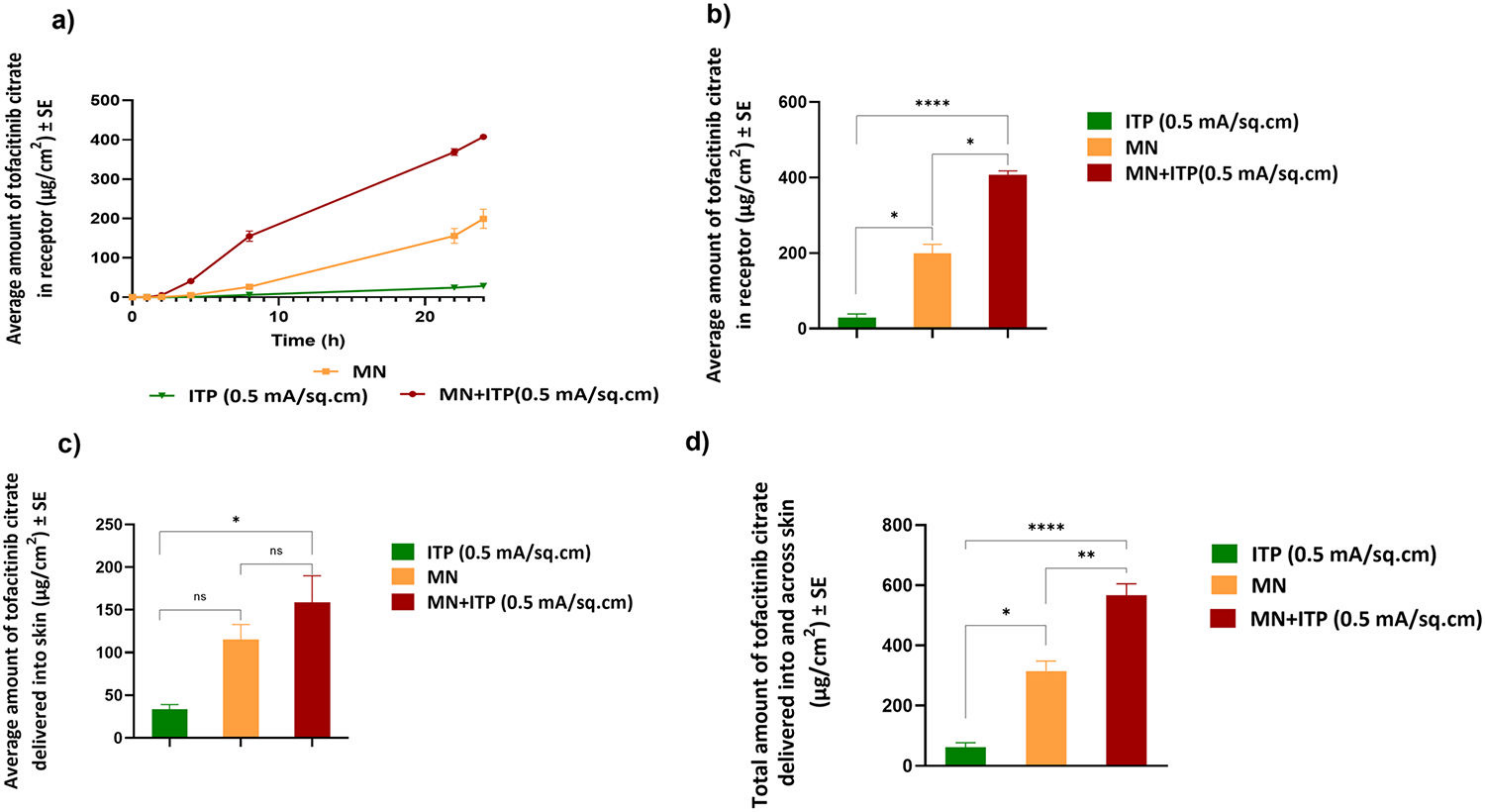
Comparative delivery: (a) Permeation profile of tofacitinib citrate across full-thickness human skin into receptor over 24 h (b) Amount of tofacitinib citrate delivered into receptor at the end of 24 h (c) Amount of tofacitinib citrate delivered into skin at the end of 24 h (d) Total amount of tofacitinib citrate delivered into and across full-thickness human skin. Data presented as average values ± SE (n=4); *: p < 0.05, **: p < 0.01, ***: p<0.001; ****: p<0.0001 ;MN: Microneedles, ITP: Iontophoresis

The effect of combination of microneedles and iontophoresis was investigated at current density of 0.5 mA/cm^2^. The total delivery of tofacitinib citrate from a combination of microneedles and iontophoresis was 566.59±19.2 μg/cm^2^, which was significantly higher than microneedles and iontophoresis alone. Figure 7 shows comparative delivery of tofacitinib citrate amongst the groups tested. Further, the lag time for permeation of tofacitinib citrate was reduced to 1.06 h using a combination of microneedles and iontophoresis.

## Discussion

Topical and transdermal delivery of tofacitinib citrate was investigated to explore this route for the administration of tofacitinib citrate for the treatment of psoriasis and psoriatic arthritis. This route can provide sustained delivery of drugs with lesser potential systemic side effects [31], which is desired for this molecule. Additionally, this route can also facilitate localized delivery at the site of action for treatment of psoriasis. Delivery into dermis would be beneficial in this case as the pharmacological targets for tofacitinib citrate (B and T cells) are in the dermis [32]. Literature reports use of topical solution of tofacitinib citrate with promising results with 2% tofacitinib citrate ointment in Phase IIa clinical trial [33]. Other studies report topical formulations [7] and proposomes [32] of tofacitinib citrate for treatment of psoriasis indicating therapeutic effectiveness of delivery into skin. However, based on the results of studies conducted, passive permeability of tofacitinib citrate was found to be very low where no receptor delivery was observed across full-thickness human skin in 24 h with low delivery into skin. This can be due to tofacitinib citrate's ionized and hydrophilic nature, making it challenging to cross lipophilic stratum corneum. Physical enhancement techniques such as microneedles and iontophoresis applied in this study can bypass stratum corneum or enhance delivery across it thereby providing delivery at the site of action for treatment of psoriasis. Besides topical delivery, transdermal delivery of tofacitinib citrate is not explored widely. Various physical enhancement techniques have been used to enhance transdermal delivery of hydrophilic molecules in past years [12, 34]. Thus, we investigated microneedles and iontophoresis alone and in combination, as mentioned earlier, to see the effect of these physical enhancement technologies on delivery of tofacitinib citrate.

**Fig. 8:**
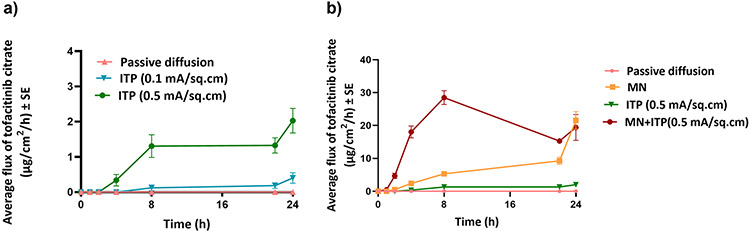
(a) Flux profile for iontophoretic delivery of tofacitinib citrate across full-thickness human skin (b) Flux profile for delivery of tofacitinib citrate across full-thickness human skin comparison between all groups. Data presented as average values ± SE (n=4); MN: Microneedles, ITP: Iontophoresis

The fabricated PVA-CMC microneedles successfully microporated skin. Skin characterization studies performed verified formation of micropores. Dye binding study conducted using methylene blue confirmed hydrophilicity of micropores as the dye was selectively taken up by hydrophilic micropores where intact lipophilic stratum corneum remained unstained. Histological sections of skin treated with microneedles and SEM images of skin treated with microneedles showed formation of micropores as well. The depth of microchannel created by microneedles was determined using confocal microscopy studies. Fabricated microneedles having a length of approximately 395 μm created microchannels 120 μm deep, consistent with results of our previous microneedle fabrication studies [20]. The fabricated microneedles created microchannels, bypassing stratum corneum and epidermis, allowing diffusion of hydrophilic tofacitinib citrate, which resulted in a 28-fold increase in delivery of tofacitinib citrate as compared to passive diffusion. A recent study conducted using dissolving microneedles showed a similar enhancement in the delivery of tofacitinib citrate across porcine skin [26], demonstrating the effect of microporation. The flux profile of tofacitinib citrate across microporated skin showed a gradual increase over time (Fig. 8b).

Iontophoresis, as mentioned earlier, aids delivery of neutral or charged molecules wherein electroosmosis and electrorepulsion are the two mechanisms at play [21]. The polar and water-soluble nature of tofacitinib citrate makes it a good candidate for iontophoresis. Moreover, as at pH 7.4 (formulation pH), tofacitinib citrate is positively charged, anodal iontophoresis was carried out. The effect of current density on iontophoretic delivery of tofacitinib citrate was investigated where an increase in current density led to increased delivery of tofacitinib citrate into and across skin. This is because the transport pathways are current dependent, causing increase in delivery with increase in current seen in other previous studies [35]. The flux profile for iontophoretic delivery of tofacitinib citrate (Fig. 8a) showed a continuous increase even after the current termination, which can be due to changes in the electrical properties of the stratum corneum or due to possible formation of drug depot in the skin. A higher current density also led to a higher average flux at every time point.

In comparison, microneedles alone delivered a significantly higher total amount of tofacitinib citrate than iontophoresis (0.5 mA/cm^2^) (Fig. 7d). A similar trend was observed previously where *in vitro* delivery of methotrexate via microporation was higher than iontophoresis [36]. However, other studies suggest higher delivery of drugs via iontophoresis [15, 18]. These differences can be attributed to differences in drug physicochemical properties, including its charge, size, lipophilicity, and other formulation factors such as pH and the presence of other competitor ions that affect its delivery via microporation and iontophoresis [21].

As iontophoresis acts on the drug molecule itself while microporation works by altering structure of skin layers, a combination of microneedles and iontophoresis was tested to elucidate its effect on delivery of tofacitinib citrate. The total delivery of tofacitinib citrate using a combination of microneedles and iontophoresis was significantly higher than microneedles or iontophoresis alone, demonstrating a synergistic effect. Literature reports a similar synergistic effect observed for delivery of prochlorperazine edisylate [37], methotrexate [38], and fluorescein isothiocyanate dextrans [39]. As seen from the flux profile (Fig. 8b), the combination of microneedles and iontophoresis showed significant enhancement in transdermal flux compared to microneedles and iontophoresis alone. Moreover, for the combination of microneedles and iontophoresis, the flux profile showed a continuous increase until 8 h, followed by a sharp decrease until 22 h. This suggested possible modulated delivery of tofacitinib citrate using a combination of microneedles and iontophoresis.

Considering the total delivery achieved in this study, a therapeutic dose of tofacitinib citrate was not achievable with passive diffusion (considering a maximum patch size of 50 cm^2^). Amongst microneedles and iontophoresis, the therapeutic dose was achievable using microneedles alone. Although iontophoresis alone was not enough to achieve the therapeutic dose, when combined with microneedles it helped deliver a therapeutic dose of tofacitinib citrate with reduced patch size (Table 1).

Apart from target delivery, lag time is an important aspect in transdermal delivery, which is the time required for the drug to permeate across skin to reach systemic circulation. A shorter lag time is preferred due to faster availability of drug at the site of action. As seen from the results, there was no receptor delivery of tofacitinib citrate across full-thickness human skin via passive diffusion in 24 h, suggesting the use of enhancement technologies to deliver the drug. Microneedles and iontophoresis alone enabled delivery of tofacitinib citrate with a reduced lag time of approximately 3.7 h. The combination further reduced lag time to 1.1 h, suggesting faster onset of action.

In addition to investigating the individual and combination effects of microneedle and iontophoresis on the delivery of tofacitinib citrate, the experimental data presented here can be used in calibration and validation of advanced computational models of transdermal drug delivery that are being developed in collaboration [40]. The modeling and simulation capabilities can extend the current state of art to investigate the delivery of the drug to the target area, distribution and residence times at the target.

Overall, these results suggest that microneedles and iontophoresis are promising technologies for transdermal administration of tofacitinib citrate. While microneedles can provide a pain-free mode of administration, iontophoresis can be coupled for faster delivery.

## Conclusion

Physical enhancement techniques, namely microneedles and iontophoresis, enhanced permeation of tofacitinib citrate into and across human skin as compared to passive diffusion. Among the two, microporation by microneedles was found to be more effective than iontophoresis to deliver more tofacitinib citrate with a decrease in lag time. However, when combined with iontophoresis, microneedles delivered significantly higher amounts than alone, demonstrating a synergistic effect. Furthermore, the combination of microneedles and iontophoresis showed reduced lag time. Thus, this study demonstrates the potential of delivering a therapeutic dose of tofacitinib citrate via transdermal route.

## Figures and Tables

**Table 1:** Projected patch size for delivery of tofacitinib citrate

Group	Amount of tofacitinib citrate delivered per unit area (μg/cm^2^)	Patch size required to achieve therapeutic dose (cm^2^)	Feasibility of patch
Passive diffusion	11.04	1340	x
Microneedles	314.7	47	✓
Iontophoresis	62.07	238	x
Microneedle + Iontophoresis	566.59	26.12	✓

## Abbreviations

JAK: Janus kinase
PVA: Polyvinyl alcohol
CMC: Carboxymethyl cellulose
SEM: Scanning electron microscopy
PBS: Phosphate buffered saline
IVPT: *In vitro* permeation testing
CLSM: Confocal laser scanning microscopy
